# Development of a miniature bioreactor model to study the impact of pH and DOT fluctuations on CHO cell culture performance as a tool to understanding heterogeneity effects at large‐scale

**DOI:** 10.1002/btpr.3264

**Published:** 2022-05-07

**Authors:** Roman Zakrzewski, Kenneth Lee, Gary J. Lye

**Affiliations:** ^1^ The Advanced Centre for Biochemical Engineering, Department of Biochemical Engineering University College London London UK; ^2^ Biopharmaceutical Development: Bioprocess Technologies and Engineering AstraZeneca Gaithersburg USA

**Keywords:** CHO cell culture, heterogeneity, miniature bioreactor, mixing, scale‐down

## Abstract

Understanding the impact of spatial heterogeneities that are known to occur in large‐scale cell culture bioreactors remains a significant challenge. This work presents a novel methodology for mimicking the effects of pH and dissolved oxygen heterogeneities on Chinese hamster ovary (CHO) cell culture performance and antibody quality characteristics, using an automated miniature bioreactor system. Cultures of 4 different cell lines, expressing 3 IgG molecules and one fusion protein, were exposed to repeated pH and dissolved oxygen tension (DOT) fluctuations between pH 7.0–7.5 and DOT 10%–30%, respectively, for durations of 15, 30, and 60 min. Fluctuations in pH had a minimal impact on growth, productivity, and product quality although some changes in lactate metabolism were observed. DOT fluctuations were found to have a more significant impact; a 35% decrease in cell growth and product titre was observed in the fastest growing cell line tested, while all cell lines exhibited a significant increase in lactate accumulation. Product quality analysis yielded varied results; two cell lines showed an increase in the G0F glycan and decrease in G1F, G2F, and Man5; however, another line showed the opposite trend. The study suggests that the response of CHO cells to the effects of fluctuating culture conditions is cell line specific and that higher growing cell lines are most impacted. The miniature bioreactor system described in this work therefore provides a platform for use during early stage cell culture process development to identify cell lines that may be adversely impacted by the pH and DOT heterogeneities encountered on scale‐up. This experimental data can be combined with computational modeling approaches to predict overall cell culture performance in large‐scale bioreactors.

## 1 INTRODUCTION

Scaling up bioprocesses from bench to production‐scale is crucial in the development of biopharmaceutical products. Small‐scale bioreactor models allow for time and cost‐efficient development and optimization of cell culture processes. During initial cell line selection, miniature bioreactors within the microliter and milliliter scales are used for high‐throughput experimentation to reduce development time and costs.^1^ Cultures are then typically scaled up to bioreactors with volumes between 1 and 50 L. Once a robust operating window has been established the process is finally scaled up to production‐scale vessels. These can range from single‐use bioreactors, up to 3000 L, to larger stainless steel vessels up to 25,000 L.^2^ There are a variety of scale‐up approaches^3^, ^4^, ^5^ which become increasingly complex and challenging to implement with scale; larger vessels are known to be less homogeneous due to longer mixing times.^6^, ^7^ The lower agitation and aeration rates used in large‐scale cell culture vessels causes fluctuations, or gradients, in temperature, dissolved oxygen concentration, and pH.^8^, ^9^

In the case of microbial fermentations, a number of publications have described the effects of heterogeneous conditions caused by poor mixing.^10^, ^11^, ^12^, ^13^ Similar studies on mammalian cell cultures are rare. As cell densities increase in industrial cell culture processes, heterogeneities are considered more likely and a number of pioneering studies have begun to address this issue.^14^, ^15^ One challenge in studying heterogeneities in large‐scale vessels is identifying and characterizing them experimentally. Most production‐scale vessels are located in commercial good manufacturing practice (GMP) facilities and hence experimentation is difficult due to the associated costs, availability, as well as the limited numbers of probes installed along the length of the bioreactor. Of the few studies available, Xing et al^16^ reported mixing times of over 100 s in a 5 m^3^ vessel and Lara et al^15^ reported higher mixing times of 120–360 s in a 12 m^3^ stirred tank reactor (STR). Others have shown that in vessels up to 8000 L poor mixing can lead to high pH excursions after base addition.^17^ Recently, a study using a transparent 15,000 L bioreactor has shown that correlations assuming a constant dimensionless mixing time are not valid at that scale; deviations up to 20% during single phase operation were reported. For two‐phase, gas–liquid, operation the authors were able to visualize temporal and spatial heterogeneities and showed that the dispersed gas phase had a strong influence on liquid mixing time.^18^

Despite the few examples of large‐scale bioreactor studies in literature, detailed and reliable data is still missing.^19^ This leaves gaps in knowledge around the true nature of mixing regimes within these large vessels. An alternative to experimental studies is the use of computational fluid dynamic (CFD) models to identify phenomena that impact scale‐up. Detecting small perturbations in pH or DOT can be difficult with physical probes but is relatively easy using computational methods. CFD has been used to identify and characterize heterogeneities and model their effects on the performance of microbial cultures through coupled kinetic or metabolic models.^20^, ^21^, ^22^, ^23^, ^24^, ^25^ It remains a challenge to validate the impact of these heterogeneities and very little work on modeling large scale cell culture performance is available.

A number of scale‐down approaches have been developed to study the effects of heterogeneities on mammalian cell culture experimentally. In cell culture the addition of base, typically sodium hydroxide or sodium bicarbonate, is used for pH control and these additions can give rise to regions of high pH around the “addition zone.”^17^ Experimental scale‐down models have been designed to mimic this and are typically characterized as single or multi‐compartment models. In a single compartment model, a parameter (for example pH) is fluctuated throughout the whole vessel to simulate heterogeneities at large scale. In a multi‐compartment model the fluctuation is introduced in a smaller bioreactor, either a STR or plug flow reactor (PFR), which represents the “addition zone.” This reactor is connected to a larger STR which represents the “bulk” or well‐mixed fraction of the production‐scale bioreactor and the culture fluid is constantly circulated through both vessels. This is considered to provide the most accurate representation of what cells may experience in a heterogeneous large‐scale vessel. Paul and Herwig^26^ provide a review of different scale‐down models for cell culture applications. Design considerations to be addressed when developing multi‐compartment scale‐down models include:^27^ pH fluctuation amplitude, fluctuation frequency, fluctuation duration (or residence time within base addition zone), volume of base addition zone, and mixing time.

Several groups have developed scale‐down compartment models to investigate heterogeneity effects.^28^, ^29^, ^30^, ^31^, ^32^, ^33^ Their approaches have two key differences: (i) whether the perturbation is a single shift or multiple shifts (i.e., the frequency of perturbation), and (ii) the volume of culture exposed to the fluctuation. To address perturbation frequency Osman et al^29^, ^34^ attempted both single and multiple perturbations and showed that regular perturbations, reaching pH 8.0 and 9.0, with durations of 200 s repeated every 6 min, had a significant impact on growth of GS‐NS0 cultures. Other studies have chosen not to fix the frequency of perturbations and only create a perturbation in the smaller bioreactor when the larger (“bulk”) STR requires base addition as would occur at large‐scale. This approach has demonstrated that pH perturbations have an effect on Chinese hamster ovary (CHO) cell growth and productivity.^27^ The effects may be cell line specific as other studies have found little effect of pH fluctuations in these compartment models.^35^ The issue of “percentage volume exposure” is difficult to address because there is very little data on the size of the base addition zone. Namdev et al.^36^ suggests that the smallest zone of heterogeneity was 0.5% of total bioreactor volume in a study of *Saccharomyces cerevisiae* fermentation in a 300 m^3^ STR. Others suggest this to be higher, around 1%^8^ or 5%.^9^, ^17^ The compartment model used by Osman et al. exposed 17% of the culture to the perturbations. Studies on the effect of DOT fluctuations are rarer but there is some evidence that DOT fluctuations affect N‐linked glycosylation but not productivity of a hybridoma cell line.^37^

Traditional compartment models using STRs and PFRs can be complex to set up and are not amenable to modern high throughput experimentation. This study presents a new approach to creation of a single‐compartment model using the ambr®15 automated miniature bioreactor system. The aim is to mimic the conditions within a small zone of a large‐scale vessel where the cells would be exposed to heterogeneities. The utility of this approach is that it will enable rapid identification of cell lines which might be vulnerable to fluctuations in pH and/or DOT. This study describes the development of a fluctuation methodology in ambr®15 bioreactors and initial evaluation of a number of cell lines with different growth characteristics. The impact of pH and DOT fluctuations on growth and productivity as well as product quality was assessed. The data obtained from these scaled‐down studies could be fed into computational population balance models which could then predict overall behavior of the cell lines at large‐scale as has been demonstrated with microbial fermentations.^38^, ^39^, ^40^, ^41^

## 2 MATERIALS AND METHODS

### 2.1 Cell line and inoculum preparation

Four recombinant GS‐CHO cell lines were used in this study: AZCL_1, AZCL_3, AZCL_4, and AZCL_5 from AstraZeneca. All cell lines were developed in proprietary media and produce monoclonal antibodies (MAbs). AZCL_1 produces a bispecific fab fragment fusion antibody and the other cell lines produce different IgG_1_ MAbs. Prior to bioreactor inoculation a vial of the cell line stock was thawed and cultured aseptically in a 250 ml shake flask in a shaken incubator (SANYO E&E Europe BV, Etten‐Leur, The Netherlands) at 140 rpm, 5% vol/vol CO_2_, and 36.5°C. The culture was passaged every 3–4 days until used for bioreactor inoculation.

### 2.2 Bioreactor setup and operation

The ambr®15 system (Sartorius Stedim Biotech, Royston, UK) is widely used in industry as a tool for high‐throughput process development and is described in detail elsewhere.^42^, ^43^, ^44^ Each ambr®15 vessel is equipped with one pitched blade impeller, a sparge tube, and sensors to monitor and control pH, dissolved oxygen tension (DOT) and temperature. Set points can be changed independently for each bioreactor and are maintained via PI control. The ambr®15 vessels were inoculated at a seeding density of 0.7 × 10^6^ cells ml^−1^ in an initial volume of 13.5 ml. The temperature of each vessel was controlled at 35.5 ± 0.5°C. The pH was controlled to a set point of 7.0 ± 0.1 using CO_2_ (max flowrate 1.23 ml min^−1^) and 1 M sodium bicarbonate. DOT was controlled at 50% of air saturation using oxygen. These set points were used for all experiments unless otherwise stated, that is, when conducting cultures with fluctuating pH or DOT conditions as discussed later. Pure nitrogen was sparged at a constant rate of 0.15 or 1.00 ml min^−1^. A solution of 0.5% vol/vol Antifoam C (Sigma Aldrich, Germany) was added to each vessel at an average rate of 20 μl per day. Proprietary media and feeds were used. Nutrient feeds were added periodically and glucose was maintained with daily additions of 500 g L^−1^ glucose stock solution.

### 2.3 Cell culture analytics

Daily measurements of metabolite concentrations (glucose and lactate) were made using a YSI 2900 analyzer (YSI Life Sciences, Yellow Springs, USA). All other measurements were made every 2 days. Viable cell density and viability measurements were made using a ViCell XR (Beckman Coulter, High Wycombe, UK) which uses the trypan blue dye exclusion method. Offline pH, pO_2_ and pCO_2_ measurements were also taken every 2 days using an ABL90 Flex Plus (Radiometer Ltd, Crawley, UK) blood gas analyzer. Beginning on day 2, samples were also taken for antibody titre measurements. Supernatant was analyzed using the Octet (ForteBio, California, USA) which uses a protein A‐based quantification method.

### 2.4 Product quality analytics (end point)

Harvested cell culture broth (cultures end on day 14) was centrifuged and then filtered through a 0.22 μm SteriFlip vacuum filter (Merck Millipore, Massachusetts, USA) before being purified using a TECAN automated liquid handling system (TECAN, Mannedorf, Switzerland) equipped with RoboColumns containing MabSelect SuRe Protein A resin (Sigma‐Aldrich). Antibody purity and aggregation was determined using a standard HPLC‐SEC method using an Agilent 1200/1100 HPLC system (Aglient Technologies, Santa Clara, USA) with a TSKgel column (Sigma‐Aldrich). Protein fragmentation was determined using a standard capillary electrophoresis (CE‐SDS) method with a Beckman PA800 Plus instrument (Beckman Coulter). Glycan profiles were obtained using a semi‐quantitative method of reversed phase HPLC (RP‐HPLC) combined with quadruple time‐of‐flight (QTOF) mass spectrometry (MS). Samples were reduced with dithiothreitol (DTT) and glycans released using N‐Glycosidase F (PGNase F, VWR Cat#3261). Samples were injected onto an Acquity UPLC BEH300 column (Waters UK, Elstree, UK), from which the eluate was directly fed into the SYNAPT mass spectrometer (Waters UK) with electrospray ionization (ESI). The mass to charge raw data was deconvoluted to a relative mass; the largest peak in each chromatogram is always the G0F glycoform and all other glycoforms are inferred based on their mass relative to the G0F peak.

### 2.5 Derived cell‐specific parameters

Average cell‐specific glucose consumption (q_Gluc_), lactate production (q_Lac_) and protein production (q_p_) rates were determined by linear regression of cumulative production (or consumption) against the cumulative integral of viable cell density (cIVC), using the following equations:

(1) $${\mathrm{IVC}}_{t}=\left(\frac{{\mathrm{VCD}}_{t-1}+{\mathrm{VCD}}_{t}}{2}\right)\times \left(t_{t-1}-t_{t}\right),$$

(2) $$\text{cIVC}=\sum {\mathrm{IVC}}_{t},$$

where *IVC*_*t*_ is the cumulative integral of viable density at time *t* (cells‐day ml^−1^), *VCD* is the viable cell density (cells ml^−1^), and *t* is time (days). The maximum specific growth rate was determined from the highest specific growth rate of the culture calculated between measured time points using the equation below:

(3) μmax=lnVCDtVCDt−1tt−tt−1.

Glucose consumption per day, gluc_*cont*_, was calculated using the following equation:

(4) $${\text{gluc}}_{{\mathrm{con}}_{t}}=\left(\left[{\text{gluc}}_{t}\right]-\left[{\text{gluc}}_{t-1}\right]+\left[{\text{gluc}}_{\text{feed}}\right]\right),$$

where [gluc_t_] is the concentration of glucose in the culture at time *t*. Cumulative values for product concentration and gluc_cont_ were then determined and rates were calculated from data during the stationary and exponential phases of cell growth. Lactate production rates were calculated between two time periods: day 0–6 and day 6–10, in order to account for the change in lactate profile due to consumption.

### 2.6 Statistical analysis

To test significance between certain conditions a simple two‐tailed Student's T‐test was used in MATLAB (Mathworks, USA). Conditions yielding *p* values lower than 0.05 were considered statistically significant.

## 3 RESULTS AND DISCUSSION

### 3.1 Inducing dissolved oxygen and pH fluctuations in a high throughput miniature bioreactor

Two‐compartment scale‐down models used to study large‐scale bioreactor heterogeneities have complex setups and required liter quantities of media making them time consuming and expensive to implement. The current work addresses this problem by using an automated miniature bioreactor platform to mimic just a portion of a larger scale vessel, representing the heterogeneous environment that a small volume of cells is exposed to. The simulation of heterogeneity is achieved by introducing programmed pH and DOT fluctuations in a well‐mixed miniature bioreactor. The pH and DOT set points are changed at fixed regular intervals and durations, similar to the approach taken by Osman et al.^29^ Using miniature bioreactors to study fluctuating heterogeneities is a novel approach; the closest reported work is that of Jiang et al.^45^ who used the larger ambr®250 reactor to study single pH excursions. In this work we use the ambr®15 (an almost 14‐fold decrease in scale) to study more frequent fluctuations throughout the culture duration.

**FIGURE 1:**
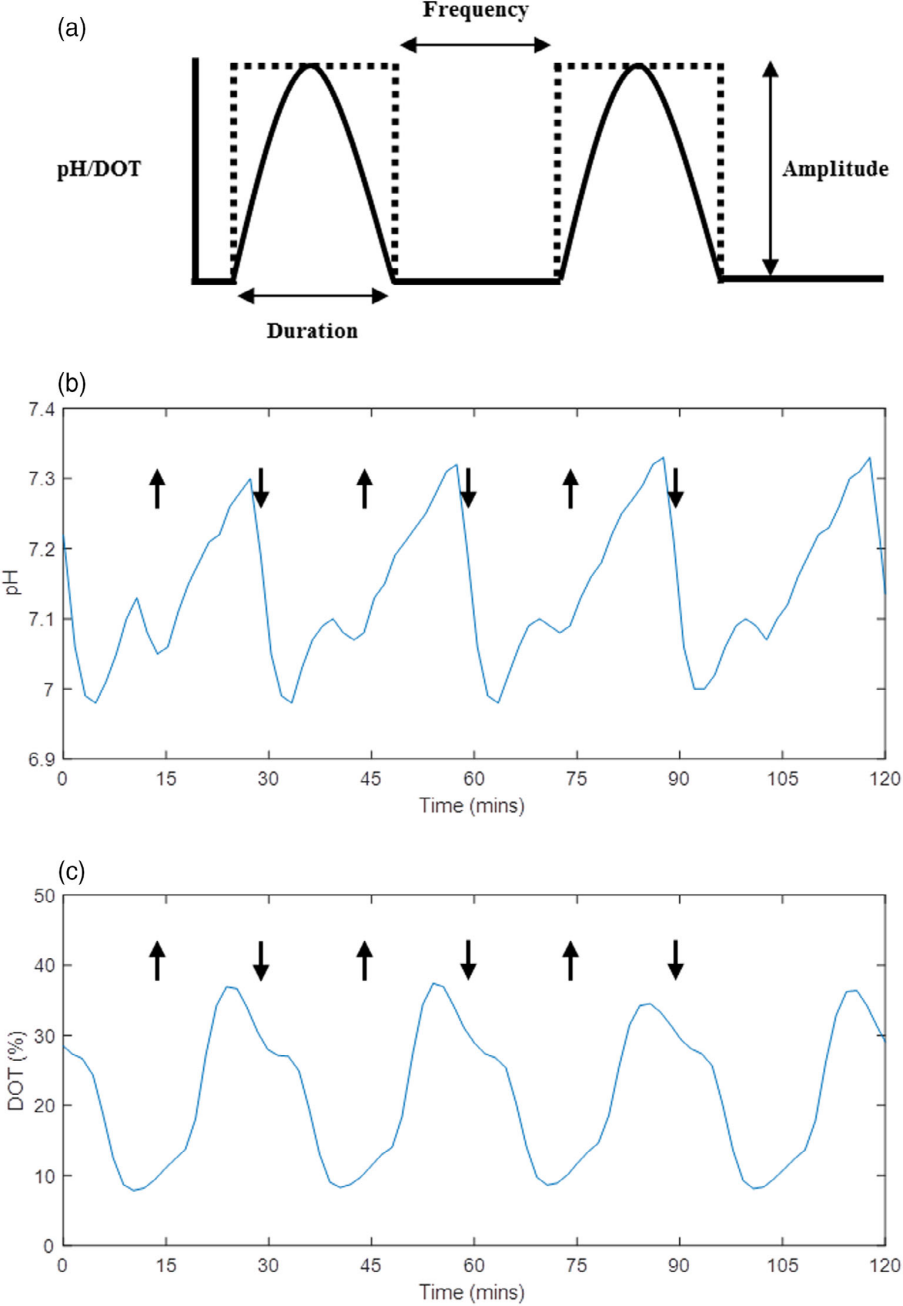
Examples of “ideal” and “real” fluctuation profiles. (a) Shows an illustration of the how the fluctuations are programmed; dotted lines represent step changes in set points (pH or DOT) and solid lines represent the “ideal” profile caused by the step change. (b) and (c) Are 2 h samples of “real” pH and DOT profiles, respectively, from two separate ambr®15 vessels, where pH and DOT were programmed to fluctuate during a fed‐batch culture of AZCL_3 between pH 7–7.5 and DOT 10%–30%, respectively. The arrows highlight where a change in set point was programmed. Both profiles (b) and (c) show fluctuations of 15 min duration and frequency. DOT, dissolved oxygen tension

Initial work focused on establishing a robust experimental methodology for creating fluctuations in the ambr®15. Figure 1a shows an example of “ideal fluctuations” and how duration, frequency and amplitude are defined. The frequency refers to how often a step change in pH or DOT is programmed. In all experiments presented in this study the duration and frequency of a fluctuation were always the same and hence the terms are used interchangeably. To create the fluctuations the bioreactor set‐points were programmed to change periodically between pH 7.0–7.5 or DOT 10%–30%.

The method for achieving fluctuations in the ambr®15 was transferred from preliminary studies using another miniature bioreactor system; the micro24® (Pall Life Sciences, Portsmouth, UK) (data not shown).^46^ When programming fluctuations on the ambr®15, conditions were kept as similar as possible to established cell culture protocols. Standard media, feeds, base (1 M sodium bicarbonate) and other additions were used in all cultures. Preliminary studies, however, highlighted the importance of gas composition and flowrate when inducing fast and frequent purturbations. To achieve the required fluctuations pure nitrogen was used as a purge gas at a higher flowrate than normal to induce quicker CO_2_ or O_2_ stripping and therefore drop pH or DOT more rapidly. Fluctuations were mainly driven by manipulating the controller settings; increasing both proportional (P) and integral (I) components to induce a more aggressive response from the controller, hence creating the fluctuations. Due to the nature of PI control in the ambr®15, the pH and DOT signals exhibited over and under‐shoot as can be seen in Figure 1b,c, respectively. These figures focus in on a series of four repeat fluctuations, showing that the fluctuations achieved were very reproducible. Maintaining the fluctuations over the full course of a cell culture (from day 2 to 14), is more challenging because culture conditions are affected by many factors including media additions to the bioreactors and differing lactate production by the cells.

**FIGURE 2:**
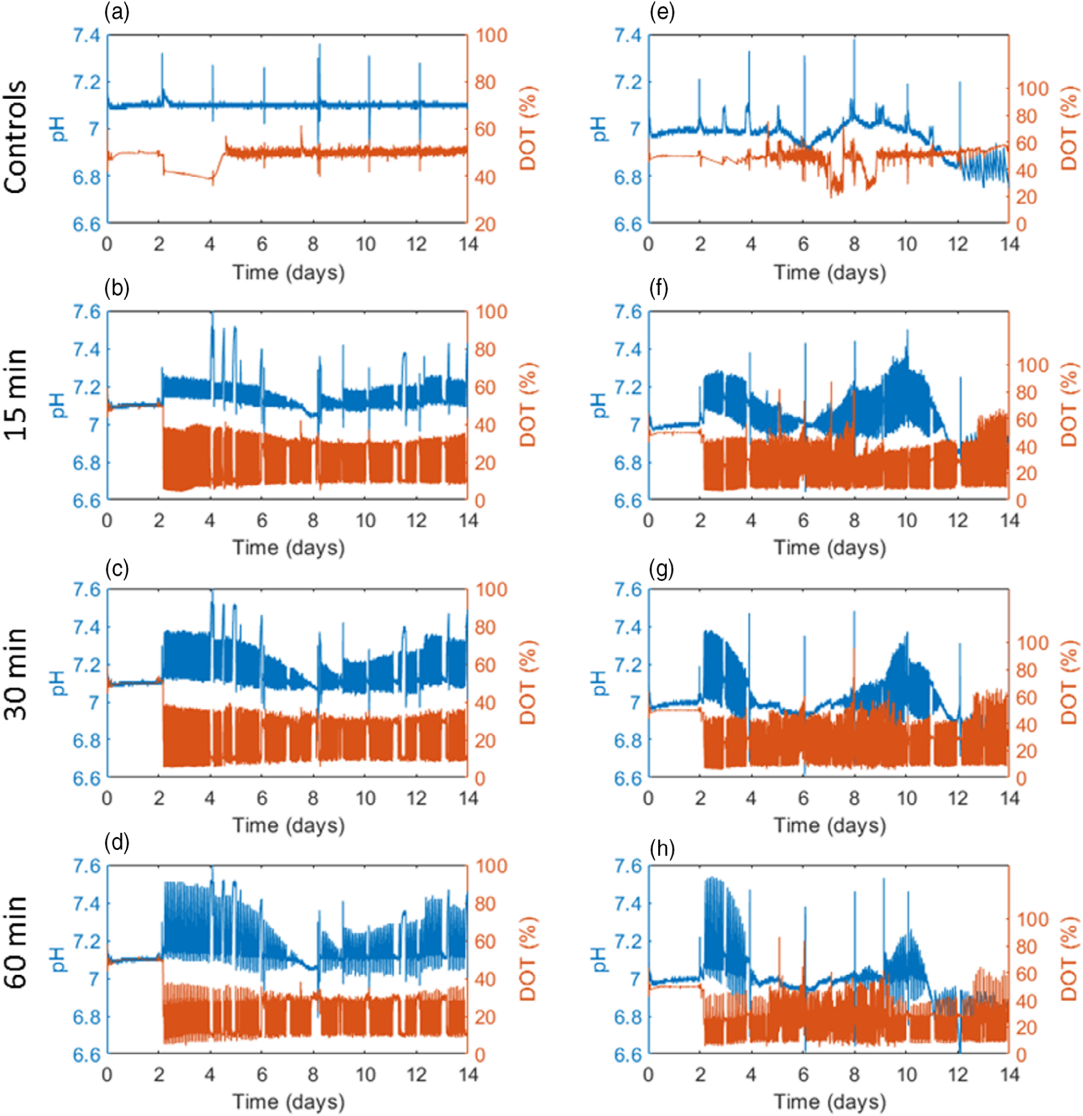
Overall fluctuation profiles in fed‐batch CHO cultures. Representative examples of typical pH and DOT profiles of individual vessels from a fed‐batch culture of AZCL_1 (left‐hand column) and AZCL_3 (right‐hand column), comparing control (a and e) and fluctuated vessels. The frequency and duration of the fluctuations were set at 15 (b and f), 30 (c and g), and 60 (d and h) minutes. The range of fluctuations programmed were pH 7.0–7.5 and DOT 10%–30%. CHO, Chinese hamster ovary; DOT, dissolved oxygen tension

Figure 2 shows the pH and DOT profiles achieved across the entire duration of fed‐batch cultures with two cell lines subjected to fluctuations; one growing to “low” peak VCD (AZCL_1) and the other to “high” peak VCD (AZCL_3). The cell lines are described in a later section. Figure 2a,e shows typical profiles of non‐fluctuated control bioreactors in the ambr®15 for AZCL_1 and AZCL_3 cultures respectively. The controls were fixed at pH 7.0 ± 0.1 and DOT 30 or 50%. The higher DOT control set‐point of 50% is typically used in ambr®15 processes to ensure oxygen is not rate limiting. The transient deviations seen in these control bioreactors every 2 days correspond to nutrient feed addition when pH control is temporarily delayed due the robotic arm of the ambr®15 being occupied. Figure 2b–d show typical pH and DOT profiles of bioreactors from AZCL_1 cultures with programmed fluctuations between pH 7–7.5 and DOT 10–30%, with a frequency of 15, 30, and 60 min, respectively. Figure 2f,g,h show the equivalent for AZCL_3 cultures.

The amplitude of the fluctuations achieved at the three frequencies were lower than programmed and many cultures struggled to reach pH 7.5. Table 1 shows the maximum range of pH and DOT fluctuations achieved during these cultures. As the duration of the fluctuations was increased from 15 to 60 min, the amplitude of the pH fluctuations improved, as seen in Figure 2. The pH is increased by stopping CO_2_ flow into the bioreactor, allowing the nitrogen to strip the CO_2_ and adding bicarbonate base to raise the pH. Longer durations give the system more time to raise the pH. This difference between the programmed and actual range is likely due to the nature of PI controls of the ambr®15, the buffering capacity of the cell culture media and lactate accumulation within the medium.

**TABLE 1:** Summary of the general range of fluctuation amplitudes achieved during ambr®15 cultures of AZCL_1 and AZCL_3 at three different programmed fluctuation frequencies: 15, 30, and 60 min. Values based on fluctuations indicated in Figure 2

Programmed Fluctuation	Frequency (min)	Cell Line AZCL_1	Cell Line AZCL_3
pH	15	7.05–7.25	6.95–7.30
	30	7.05–3.40	6.95–7.40
	60	7.05–7.50	6.95–7.50
DOT	15	10–35	10–50
	30	10–35	10–50
	60	10–35	10–50

Abbreviation: DOT, dissolved oxygen tension.

**FIGURE 3:**
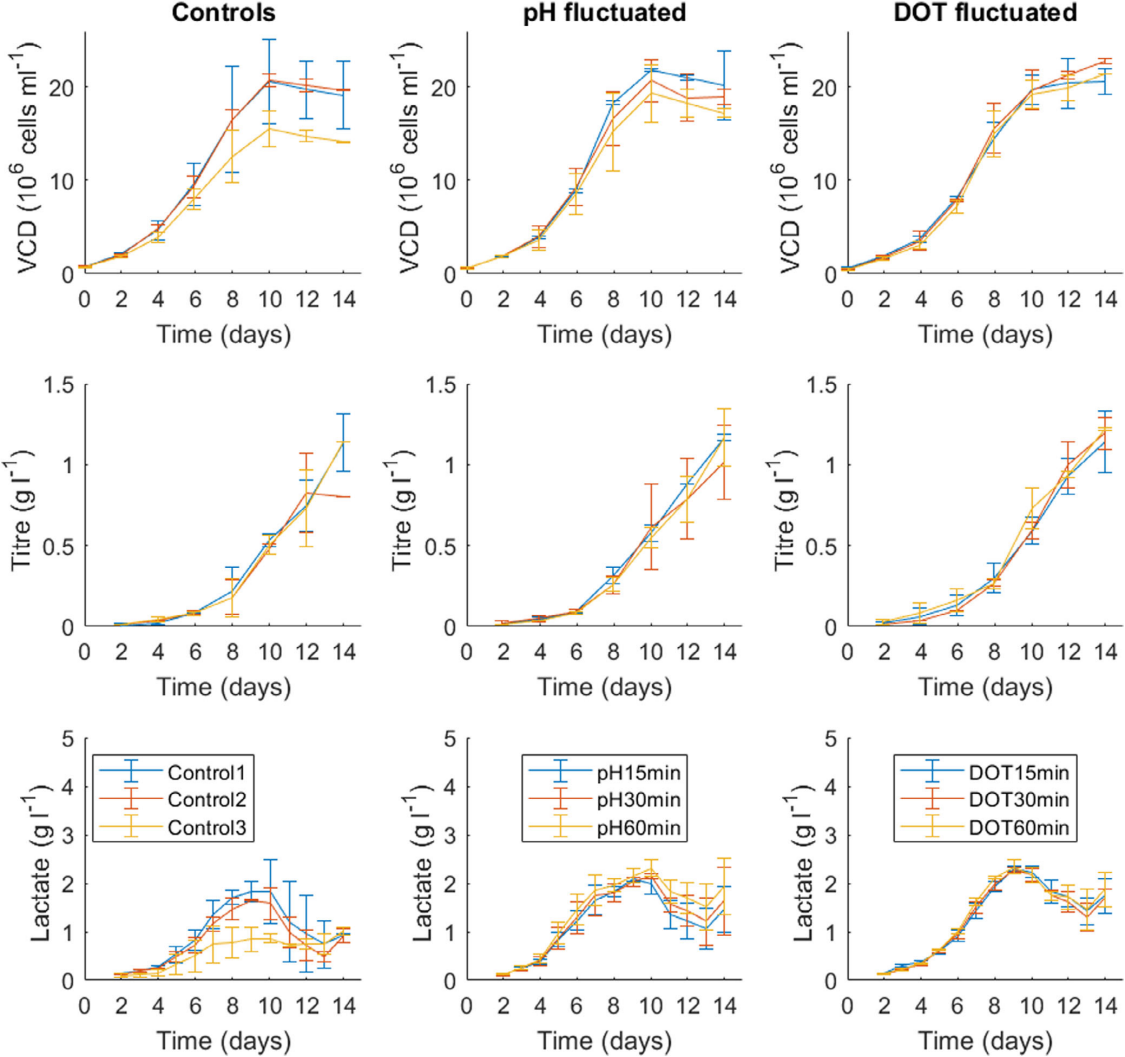
The performance of AZCL_1 (“low” growing cell line) ambr®15 cultures subjected to pH (middle column) and DOT (right column) fluctuations at frequencies of 15, 30, and 60 min. Cell growth (row 1), antibody titre (row 2) and lactate concentration (row 3) data were gathered from duplicate or triplicate vessels and error bars represent 1 SD from the mean. All controls (left column) were set to 50% DOT and pH 7.1 but varied in PI settings and N_2_ flow rates as described in Table 2. DOT, dissolved oxygen tension

**FIGURE 4:**
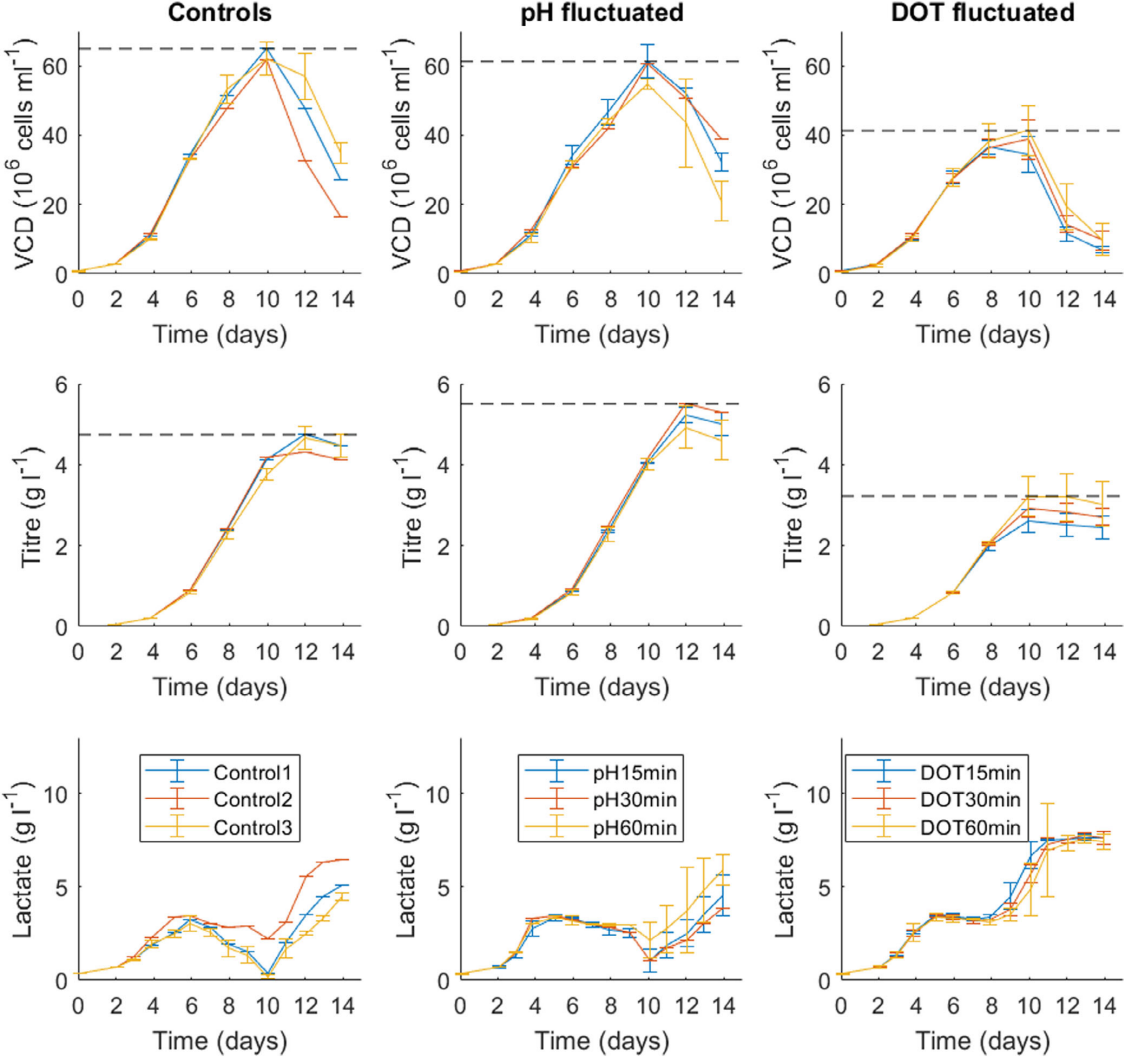
The performance of AZCL_3 (“high” growing cell line) ambr®15 cultures subjected to pH (middle column) and DOT (right column) fluctuations (Table 1) at frequencies of 15, 30, and 60 min with respect to cell growth (row 1), productivity (row 2), and lactate concentration (row 3). All controls were run at pH 7.1. Control 1: new PID setpoints, higher gas flow rate, DOT 50%. Control 2: original PID setpoints, higher gas flow rate, DOT 30%. Control 3: original PID setpoints and slower (old) gas flowrate, DOT 50% (Table 2). pH‐fluctuated vessels were held at 50% DOT. Data was gathered in duplicate or triplicate and the error bars represent 1 SD from the mean. The hashed horizontal lines indicate the highest “peak” values achieved in each culture. Effects of pH and DOT fluctuations on product quality. DOT, dissolved oxygen tension

The pH fluctuations were the most difficult to maintain experimentally. Around day 8 of the cultures (earlier in AZCL_3 cultures) the pH fluctuations tapered off and the pH remained at the lower (optimal) set point for several hours or days before the fluctuations restarted. During this period no CO_2_ was being sparged into the system. This effect is more dramatic in AZCL_3 cultures and increases with fluctuations of longer duration (Figure 2h). This is likely due to higher lactate concentrations in the AZCL_3 cultures; lactate cannot be driven out of the culture the same way that CO_2_ can be, and this drives the pH down. At day 4 and 8 the peak lactate concentration was reached, in AZCL_1 and AZCL_3 cultures respectively, at which point lactate consumption was seen (Figures 3 and 4), allowing fluctuations to return.

In contrast, stable DOT fluctuations were achieved with all cell lines across all frequencies. This is because the DOT control system does not rely on addition of liquid and is not limited by the buffering capacity of the media, which inherently makes it more difficult to maintain a stable pH set point. The DOT fluctuations were driven by the manipulation of the O_2_ flow rate; the oxygen consumption rate of the cells caused the DOT to drop even quicker. As a result, the response is much quicker than with pH and hence better fluctuations are achieved. AZCL_3 cultures grew to higher densities and therefore exhibited more noise in their DOT profiles and the fluctuation amplitudes were higher due to PI controller overshoot. In general, the pH and DOT fluctuations achieved were stable. Specific control of DOT was easier to achieve than pH. This initial work demonstrates that it is possible to programme and maintain fluctuations in the ambr®15 bioreactors throughout the course of a CHO fed‐batch culture.

### 3.2 Effect of pH and DOT fluctuations on a “low” peak VCD industrial cell line

Preliminary studies using the micro24® bioreactor indicated that the effects of fluctuations are likely to be cell line dependent.^46^ A key difference between cell lines likely to influence their response to fluctuations is their peak viable cell density. In this study two industrial cell lines were compared; one with a ‘low’ peak VCD (~21 x 10^6^ cells ml^−1^) and the other with a “high” peak VCD (~60 x 10^6^ cells ml^−1^). This section describes the investigation into the “low” growing cell line (AZCL_1).

Previous studies investigating the effects of pH heterogeneity have typically introduced fluctuations by adding base either on a fixed daily basis^45^ or at a frequency that matches fluctuations reported in large‐scale vessels.^27^, ^35^ This approach of adding more base was attempted in preliminary studies^46^ and resulted in the same issue of very high osmolality (>700 mOsm kg^−1^) as encountered in the cited works. For this reason, the pH fluctuations reported here were induced by changing controller set points and PI settings, reducing excessive base addition. The ambr®15 automatically added base if the pH fell below 6.9. In the case of AZCL_1 lactate build‐up during the culture did not exceed the buffering capacity of the medium hence no additional base was used. However, without base addition, the amplitude of the pH fluctuations was limited.

The pH and DOT fluctuations achieved experimentally are shown in the left column of Figure 2. Due to the lower cell density of AZCL_1 cultures, the pH fluctuations were relatively stable throughout the culture. A trade off exists when optimizing PI settings and gas flowrates to induce fluctuations; fluctuations may be larger and quicker with more aggressive gas flowrates but this in turn may negatively affect the cell culture^7^ and overshadow the effects of the fluctuations themselves. One obvious consequence of high‐gas flowrates would be the increased usage of antifoam. To exclude any negative effects of changing the gas flowrates and PI settings, “control” bioreactors were set up to compare standard conditions with the newly proposed conditions. Table 2 summarizes the controls that were run in duplicate. Control 3 is the original (lower) N_2_ purge gas flow rate and PI settings. Controls 1 and 2 have the “new” set up; the former using all the new conditions, that is, higher N_2_ flowrate and PI values and the latter keeps the original PI values but has a higher N_2_ flow rate. All vessels were cultured with the standard (lower) PI and N_2_ flowrates until day 2, at which point fluctuations were initiated.

**TABLE 2:** Summary of control parameters used for AZCL_1 and AZCL_3 cultures with programmed fluctuations. Table shows the DOT and pH set points (s.p) and N_2_ gas flowrates. The original *p* and I values based on standard methods were −80 and −0.4 respectively, and all vessels were cultured with these values as well as the lower (0.15 ml min^−1^) N_2_ flowrate until day 2, when fluctuations began

Control	DOT % s.p	pH s.p	*p* value	I value	N_2_ (ml min^−1^)
AZCL_1					
C1	50	7.1	−200	−2.0	1.0
C2	50	7.1	−80	−0.4	1.0
C3	50	7.1	−80	−0.4	0.15
AZCL_3					
C1	50	7.0	−200	−2.0	1.0
C2	30	7.0	−200	−2.0	1.0
C3	50	7.0	−80	−0.4	0.15

Abbreviation: DOT, dissolved oxygen tension.

Fluctuation frequencies of 15, 30, and 60 min were investigated in triplicate. Figure 3 compares the performance of control vessels and those exposed to pH and DOT fluctuations with respect to cell growth, antibody productivity, and lactate concentration. The left column in Figure 3 shows the performance of the three control cultures where no fluctuations were introduced. All of the control cultures performed similarly to previous AZCL_1 cultures run at 5 L scale achieving a peak VCD of around 19 x 10^6^ cells ml^−1^. Control 3 appears to grow 22% less than the other two controls with higher gas flowrate, peaking at only 15 x 10^6^ cells ml^−1^. This is also reflected in the lower lactate accumulation, suggesting that the lower N_2_ flowrate may be the cause of lower growth and lactate production (q_lac_) in AZCL_1 cultures. Interestingly however, the titre was not affected in the same way, meaning the q_p_ of Control 3 was higher than the two other controls (Table 3).

**TABLE 3:** Summary of specific production and consumption rates for AZCL_1 ambr®15 cultures with fluctuating pH and DOT profiles. Data was gathered from duplicate or triplicate vessels and error bars represent 1 SD from the mean. The coefficient of determination (R^2^ value) for the linear regressions were all above 0.95, except those marked with an asterix, which were in the range of 0.55–0.95

Condition		q_gluc_ (pg cells^−1^ h^−1^)	q_lac_ (day 0–6) (pg cells^−1^ h^−1^)	q_lac_ (day 6–10) (pg cells^−1^ h^−1^)	q_p_ (pg cells^−1^ h^−1^)	μ_max_ (h^−1^)
Control‐1		8.42 ± 0.9	1.42 ± 0.09	0.72 ± 0.79*	0.261 ± 0.04	0.496 ± 0.01
Control‐2		8.39 ± 0.1	1.22 ± 0.17	0.55 ± 0.37*	0.224 ± 0.02	0.52 ± 0.01
Control‐3		8.91 ± 0.12	0.98 ± 0.56	0.32 ± 0.27*	0.301 ± 0.06	0.483 ± 0.01
pH	15 min	8.28 ± 0.02	2.46 ± 0.44	0.44 ± 0.01*	0.277 ± 0.03	0.552 ± 0.06
	30 min	8.44 ± 0.33	2.53 ± 0.4	0.58 ± 0.34	0.292 ± 0.09	0.53 ± 0.03
	60 min	8.63 ± 0.72	3.09 ± 0.55	0.66 ± 0.52	0.311 ± 0.1	0.562 ± 0.08
DOT	15 min	8.42 ± 0.79	1.92 ± 0.17	0.92 ± 0.25*	0.317 ± 0.04	0.569 ± 0.11
	30 min	8.48 ± 0.49	2.1 ± 0.05	0.81 ± 0.28*	0.32 ± 0.01	0.61 ± 0.05
	60 min	8.89 ± 0.53	2.5 ± 0.16	0.79 ± 0.25*	0.388 ± 0.01	0.581 ± 0.05

Abbreviation: DOT, dissolved oxygen tension.

Comparing across the rows in Figure 3 it is clear that there is little difference in viable cell density, titre and lactate levels between control and fluctuated vessels. The viable cell density of almost all the cultures peaked at around 21 x 10^6^ cells ml^−1^, similarly with antibody titre in most cultures it peaked around 1.2 gl^−1^. The lactate profiles of the cultures were also similar; the controls have slightly lower peak lactate concentrations (particularly Control 3) below 2 gl^−1^ compared to the pH and DOT fluctuated vessels that peaked around 2.3 gl^−1^. However, this difference was found not to be statistically significant. All cultures experience a metabolic shift into lactate consumption around day 10. A slight difference in viable cell density between the 15, 30, and 60 min pH fluctuated cultures was observed; the cultures with longer fluctuation frequencies (60 min) had lower growth, however this difference was also not significant (*p* > 0.05). This difference is not seen in cultures with DOT fluctuation.

Looking at the derived culture parameters in Table 3, no effect on glucose consumption (q_gluc_) was observed. Fluctuated vessels did, however, have higher initial lactate production (q_lac_), particularly in pH‐fluctuated vessels which had lactate production rates twice as high as controls between day 0 and 6. Fluctuations appeared to also slightly increase the growth rate (μ_max_), as seen in Table 3. This matches literature observations concerning the effects of higher pH on CHO cell culture kinetics.^47^, ^48^ However, neither type of fluctuation had a significant impact on the performance of AZCL_1. These results confirm observations from preliminary studies; the effects of fluctuations appear to be cell line specific and are less likely to impact lower growing cell lines. The following section therefore describes the impact of the same fluctuations on a “high” growing cell line.

### 3.3 Effects of pH and DOT fluctuations on a “high” peak VCD industrial cell line

Higher growing cell lines require better bioreactor control as the high‐cell densities achieved can lead to high‐oxygen uptake rates and increased lactate accumulation. For this reason, high‐cell density cultures performed at large scale could be more susceptible to spatial gradients and heterogeneities.^15^ Cell line AZCL_3 grows to densities approximately three times higher than AZCL_1 and hence was used to test this hypothesis. The ambr®15 experimental set up for AZCL_3 cultures was the same as AZCL_1 cultures with the exception of Control 2 where the DOT set point was changed to 30% and the new PI settings were applied (Table 2). A clear difference between the two cell lines is that after day 10, the viability of AZCL_3 cultures decrease rapidly compared to AZCL_1 culture, as seen in Figure 4. This was expected with AZCL_3 due to its rapid growth; the cell culture quickly depletes the nutrients in the media and feeds; while lactate and other metabolite concentrations increase to detrimental levels.

As with the low‐growing cell line (AZCL_1), pH fluctuations are seen to have little effect on growth, productivity or lactate levels in AZCL_3 cultures. The control and pH‐fluctuated vessels had very similar growth profiles peaking around 62 × 10^6^ cells ml^−1^, which again match the growth profiles seen in previous 5 L cultures. The 60 min pH fluctuations did appeared to have slightly lower growth and product titre on average, compared to the other fluctuation frequencies. However, the 15 and 30 min pH‐fluctuated vessels had higher peak product titre (5.5 gL^−1^) compared to the control vessels (4.9 gL^−1^). This was a slightly surprising result although these differences were not statistically significant.

The lactate profiles of Controls 1 and 3 were similar; lactate concentration reached approximately 3 gl^−1^ on day 6 when the metabolic shift occurred and lactate consumption began. Lactate increased toward the end of the culture as the cells died. Control 2 had a slightly different profile to the other controls, particularly with regard to lactate. This control was set at 30% DOT to test whether the effects of fluctuating DOT between 10% and 30% were not simply due to the overall lower DOT (compared to 50% DOT). Control 2 had a higher initial lactate production, q_lac_, and the culture switched to consumption on day 5; but had a much lower consumption rate than the other controls (Table 4). This profile more closely matched the lactate profiles of the pH‐fluctuated vessels. This suggests that although pH fluctuations appear not to have a significant effect on growth and productivity there may be an effect on metabolic activity, which has been observed in other studies.^45^, ^47^ This is a relevant observation as it is known that increased lactate production can be an issue during scale up.^3^, ^49^

**TABLE 4:** Summary of specific production and consumption rates for AZCL_3 ambr®15 cultures with fluctuating pH and DOT profiles. Data was gathered from duplicate or triplicate vessels and error bars represent 1 SD from the mean. The coefficient of determination (R^2^ value) for the linear regressions were all above 0.95, except those marked with an asterix, which were in the range of 0.60–0.95

Condition		q_gluc_ (pg cells^−1^ h^−1^)	q_lac_ (day0‐6) (pg cells^−1^ h^−1^)	q_lac_ (day6‐10) (pg cells^−1^ h^−1^)	q_p_ (pg cells^−1^ h^−1^)	μ_max_ (h^−1^)
Control‐1		4.22 ± 0.01	1.77 ± 0.01*	−0.59 ± 0.01	0.649 ± 0.01	0.712 ± 0.01
Control‐2		4.81 ± 0.27	1.91 ± 0.08*	−0.17 ± 0.13*	0.644 ± 0.06	0.723 ± 0.02
Control‐3		4.29 ± 0.24	1.68 ± 0.3*	−0.58 ± 0.11	0.598 ± 0.01	0.713 ± 0.01
pH	15 min	4.47 ± 0.28	1.74 ± 0.06*	−0.51 ± 0.11	0.675 ± 0.04	0.757 ± 0.02
	30 min	4.57 ± 0.01	1.7 ± 0.01*	−0.52 ± 0.01	0.739 ± 0.01	0.783 ± 0.01
	60 min	4.88 ± 0.07	1.76 ± 0.04*	−0.26 ± 0.19*	0.719 ± 0.01	0.718 ± 0.03
DOT	15 min	5.8 ± 0.39	2.13 ± 0.04*	1.03 ± 0.32*	0.585 ± 0.03	0.709 ± 0.02
	30 min	5.61 ± 0.35	2.08 ± 0.01*	0.73 ± 0.25*	0.638 ± 0.01	0.721 ± 0.02
	60 min	5.45 ± 0.54	2.08 ± 0.16*	0.5 ± 0.5*	0.679 ± 0.04	0.749 ± 0.01

Abbreviation: DOT, dissolved oxygen tension.

In contrast, cultures with fluctuating DOT conditions demonstrated significant differences to control cultures in the case of the “high” growing AZCL_3 cell line. DOT‐fluctuated cultures showed a 35% decrease in growth compared to controls. This decrease impacts the productivity by the same degree; DOT‐fluctuated cultures had peak antibody titres between 2.5 and 3.1 gl^−1^; significantly lower than controls (*p* value <0.05). The specific productivity, q_p_, was not affected however (Table 4). The DOT‐fluctuated vessels had higher initial lactate production rates (q_lac [day 0−6]_) than controls, as seen in Table 4, and exhibited minimal lactate consumption. Cultures instead entered a “negative feedback loop,” where lactate accumulation induced more base addition (than controls) and caused lactate runaway, as seen in Figure 4. It appears that this increase in the lactate concentration is responsible for the observed decease in growth and productivity. The lactate profile of control 2 matched the profiles of the DOT‐fluctuated vessels, which suggests that the increased lactate accumulation may be caused by the overall lower (30%) DOT. Specific glucose consumption, q_gluc_, was also affected by DOT fluctuations; all DO‐fluctuated vessels exhibited higher q_gluc_ than controls, as seen in Table 4.

A previous study has found that fluctuating DOT in a hybridoma cell line caused higher lactate yield.^37^ These results also match other studies where a cell line producing a conjugated IgG2 was shown to have decreased product titre, increased lactate accumulation as well as lower drug‐to‐antibody (DAR) ratio after being exposed to DOT fluctuations.^50^ Other recent papers have shown that reducing the DOT, in order to mimic the DOT levels cells are expected to be exposed to at large‐scale, caused an increase in peak lactate and reduced the lactate consumption rates.^51^ They also showed that lower DOT cultures matched the profiles of 5000 L cultures and this negative impact was attributed to DOT heterogeneity, or more specifically hypoxic conditions. In addition to decreased viability the study found that lowering DOT also reduced ammonium levels, which was consistent with increased glycolysis and reduced amino acid catabolism observed in the lower DOT cultures^51^ The agreement between the findings of our small‐scale studies (15 ml) and these larger scale studies (>3 L) supports the use of the miniature bioreactor platform for studying the response of different cell lines to culture heterogeneities.

### 3.4 Effects of pH and DOT fluctuations on product quality

All cultures shown in Figures 3 and 4 were harvested on day 14, and the purified antibody was analyzed in order to evaluate the impact of fluctuating culture conditions on key product quality attributes. These attributes include protein aggregation (HPSEC), fragmentation (CGE), and N‐linked glycosylation profiles.^52^

#### 3.4.1 Aggregation and fragmentation

As discussed above, pH fluctuations had little impact on the growth or productivity of both cell lines and this was also seen in product quality data. With regard to product aggregation and fragmentation, there appeared to be more variation between triplicate vessels with fluctuated conditions however pH fluctuations did not show significant differences in either AZCL_1 or AZCL_3 cultures.^46^ With regard to aggregation, DOT fluctuations had no impact on AZCL_1, but in AZCL_3 cultures a 25% increase in high‐molecular weight (HMW) species, compared to controls, was observed across all fluctuation frequencies. There was also an increase in low‐molecular weight species (LMW) which together lead to a 2%–3% drop in overall purity in DO‐fluctuated vessels. This difference, albeit small, was significant (*p* = 0.004). There was no fragmentation observed in AZCL_3 cultures. The AZCL_1 cell line produces a bispecific fusion product, so upon reduction fragmentation is expected, although there were no significant differences between fluctuated vessels and controls. However, in both cell lines, an increase in LMW species before reduction was observed in DOT‐fluctuated vessels compared to controls. Upon reduction a similar increase was observed in the levels of the light chain (LC) fragment, suggesting that DOT‐fluctuated cultures over‐produced free‐LC. As well as the increased level of LC these cultures also exhibited a decreased level of heavy chain (HC) species.^46^

#### 3.4.2 Glycan analysis

Glycan analysis was focused on AZCL_3 cultures due to the observed impact of fluctuations on growth and productivity. The glycan profiles of all the cell lines used in this study are dominated by the bi‐antennary glycan with no terminal galactose (G0F), which makes up >68% of the overall profile. The next largest peaks are typically the galactosylated glycans G1F and G2F, which have been reported grouped and make up between 15% and 30% of the profile. High‐mannose structures (Man5) are another key glycan that is typically monitored and the cell lines had <5% of these species present.

**FIGURE 5:**
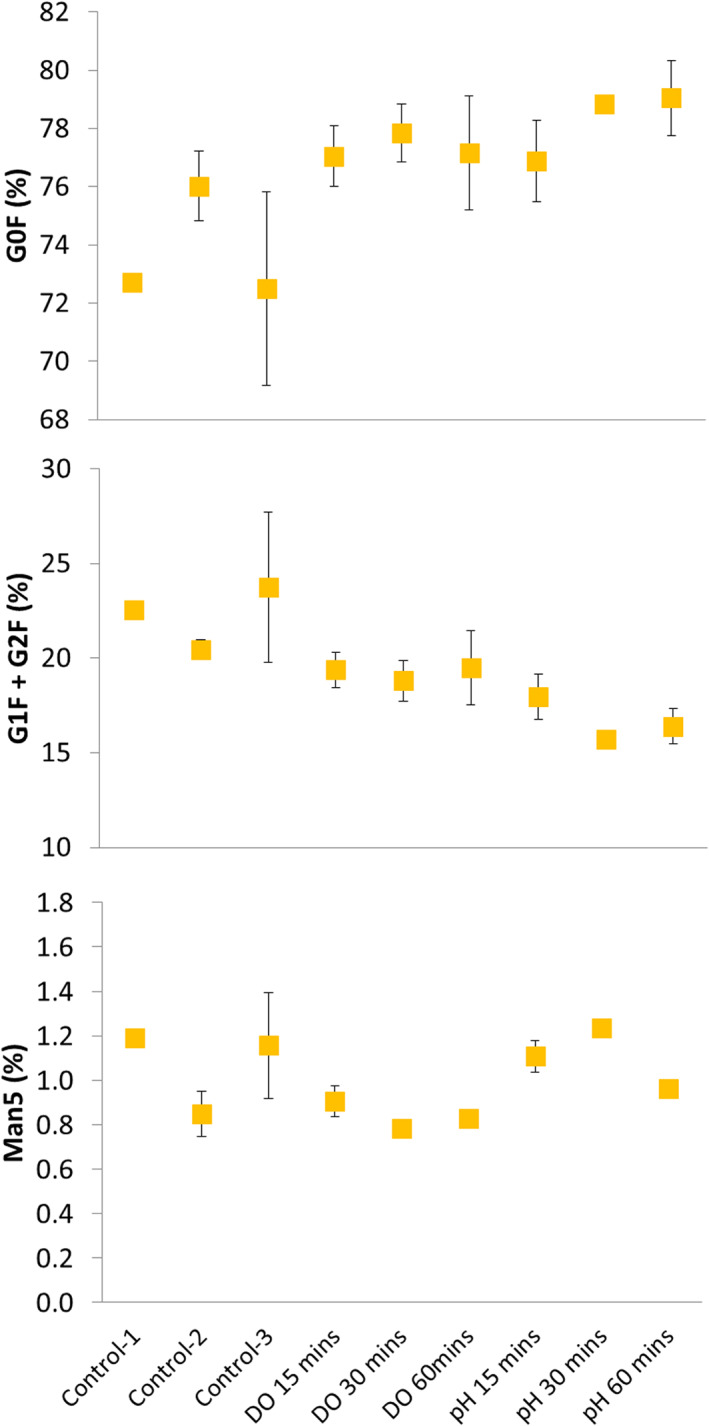
Effect of pH and DOT fluctuations at various frequencies (15, 30, and 60 min) on selected glycan structures of antibodies produced in AZCL_3 cultures (“high” growing cell line). Graph highlights the common glyans G0F, G1F, G2F, and Man 5; the levels of G1F and G2F are combined. Data is taken from harvest‐point samples from cultures shown in Figure 4. Controls are described in Table 2. Error bars represent 1 SD about the mean (*n* = 2 or 3). DOT, dissolved oxygen tension

Figure 5 shows the levels of the three major Fc glcans (G0F, G1F, G2F) and Man5 from AZCL_3 cultures, where differences between fluctuated conditions and the controls were observed. It is thought that pH can affect the rates of enzymatic reactions in the Golgi apparatus which can in turn affect glycosylation.^53^, ^54^ The pH‐fluctuated vessels were set at 50% DOT, as were the Controls 1 and 3 (Table 2) making them the most appropriate controls to compare to the pH‐fluctuated vessels. Control‐2 was set to 30% DOT and hence this is the more appropriate control to compare to the DOT‐fluctuated vessels. The pH‐fluctuated vessels had up to 11% higher levels of G0F, 12% lower levels of G1F + G2F but similar levels of Man5 compared to controls (C1 and C3). This is in contrast to the study by Jiang et al^45^ who observed higher galactosylation in pH‐fluctuated conditions. However, the impact of pH on glycosylation is not fully understood and other studies have shown similar effects to the present study. Ivarsson et al.^53^ provide a detailed discussion on various conflicting results for the effects of changing process parameters, including pH and DOT, on glycosylation (although largely under non‐fluctuating conditions).

While DOT fluctuations had a pronounced impact on cell growth (Figure 4), they had less impact on antibody glycosylation (Figure 5). Man5 levels appeared to be slightly lower in DOT‐fluctuated vessels compared to pH‐fluctuated vessels and controls 1 and 3 (by approx. 0.4%). When comparing DO‐fluctuated vessels to control 2 the differences are even smaller. This suggests that the effects seen in the DO‐fluctuated vessels may be due to the overall lower DOT that cells are exposed to in these cultures, rather than the fluctuation itself. The effects of DOT‐fluctuations observed in this study match other reports. Serrato et al^37^ showed that DOT oscillations decreased cell growth, increased glycolysis, and affected N‐linked glycosylation, but not the productivity of a MAb producing hybridoma cell line. More recent studies have attributed a reduction in titre^55^ and sialyation,^51^ in a Fc‐fusion protein producing CHO cell line, to DOT heterogeneity during scale‐up. The AZCL_3 line did not express any sialylated glycans; however, two additional cell lines were exposed to DOT fluctuations and one of these (AZCL_4) expressed low levels of sialylated glycans, which is described in the next section.

### 3.5 Effect of DOT fluctuations on other cell lines

Two additional cell lines (AZCL_4 and AZCL_5) were used to further explore the effects of DOT fluctuations. Similar experiments were set up with AZCL_5 and AZCL_4 where DOT was fluctuated between 10% and 30% in the same manner. A positive (30% DOT) and negative (10% DOT) control were used with the higher PI settings, as described in Table 2, and only 15 and 60 minute frequencies were tested. The constant N_2_ flowrate was set to 1.00 ml min^−1^ in AZCL_5 cultures and 0.15 ml min^−1^ in AZCL_4 cultures to assess if DOT fluctuations could be achieved with standard flow rates. Stable DOT fluctuations were achieved with both N_2_ flowrates. AZCL_5 and AZCL_4 cultures peaked at around 35 × 10^6^ cells ml^−1^ and 40 × 10^6^ cells ml^−1^, respectively, so they are not as low growing as AZCL_1 but not as high as AZCL_3. In both additional cell lines DO‐fluctuated cultures had lower peak VCD values and higher lactate accumulation compared to controls, but these differences were less substantial than ones observed in AZCL_3 cultures. There was no discernible difference between the negative control and DOT‐fluctuated vessels, suggesting the effects may be due to the overall lower DOT. The results with these additional cell lines support the hypothesis that higher growing cell lines are more susceptible to DOT fluctuations (or shifts in DOT set points). The effects also appear to be highly cell line specific, which is in agreement with the mixed results seen in other published studies.

**FIGURE 6:**
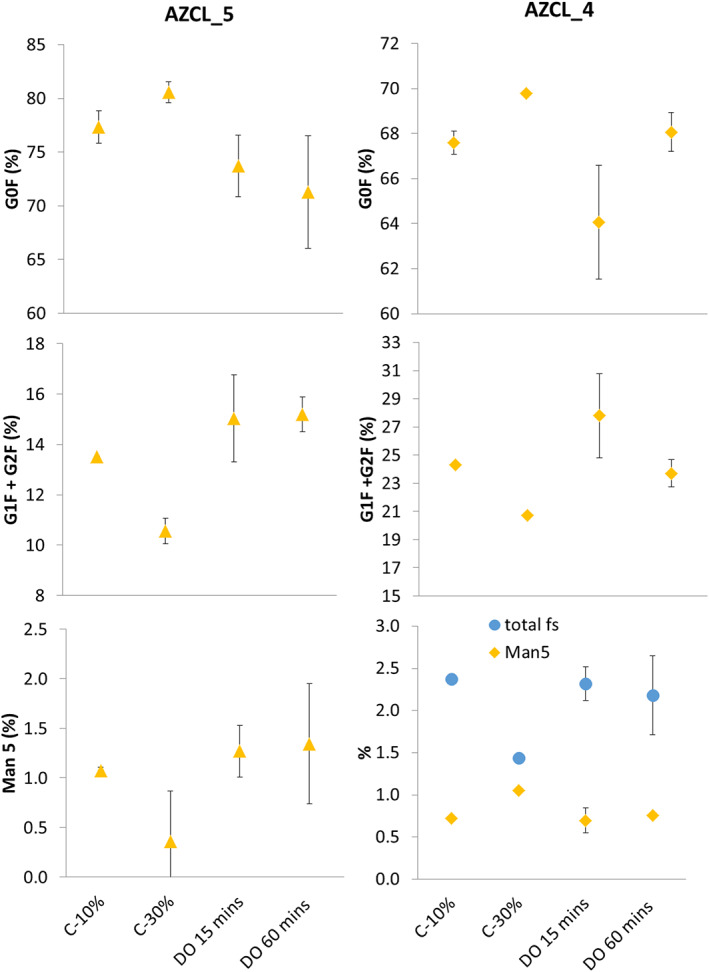
Effect of DOT fluctuations on glycosylation of antibodies produced in AZCL_4 and AZCL_5 cultures. Controls at 30% and 10% DOT were used. Fluctuation frequencies of 15 min (DO15) and 60 min (DO60) was tested. AZCL_5 cultures were run with the higher PI and N_2_ flowrate settings and AZCL_4 was run with the higher PI settings and lower N_2_ flowrate (0.15 ml min^−1^), as described in Table 2. Total fs refers to the totally percentage of sialylated glycans. Error bars represent 1 SD about the mean (*n* = 2 or 3). DOT, dissolved oxygen tension

Figure 6 shows the impact of DOT fluctuations on antibody glycosylation from AZCL_5 and AZCL_4 cultures. The effects seen on these cell lines were of a similar magnitude to AZCL_3 cultures (Figure 5), but showed slightly different trends. DOT‐fluctuated vessels show decreased levels of G0F and elevated G1F + G2F glycans compared to the 30% DOT control. Man5 levels in DOT‐fluctuated vessels appeared elevated in AZCL_5 cultures but lower in AZCL_4 cultures. These trends are also seen in the negative 10% DOT control which suggests that the DOT fluctuations are impacting the glycan profiles in a similar manner to cultures in hypoxic conditions. Many of the fluctuated cultures showed a slightly larger difference between positive 30% control than the negative control, suggesting that the fluctuations may be exacerbating the effect. The AZCL_4 IgG molecule expressed sialylated glycoforms; their levels increased slightly in DOT‐fluctuated vessels and the 10% DOT (negative) control, which partially matches the limited literature in this area.^51^ Serrato et al^37^ showed an increase in sialylated glycans in cultures with oscillating DOT of periods above 6400 s in an IgG producing hybridoma cell line. Ivarsson et al.^53^ also observed a slight increase in sialyation with a similar cell line when culturing at lower DOT set points. However, the latter study acknowledges the large variation in reported effects of DOT on glycosylation and suggests effects are cell line and product specific. The results of this study support this conclusion.

## 4 CONCLUSIONS

A novel methodology using an automated miniature bioreactor system to investigate the effects of pH and DOT fluctuations on CHO cell culture performance is presented. This provides a high throughput platform for assessing the potential impact of large‐scale bioreactor heterogeneities on different cell lines and antibody products. In the two different cell lines used to exemplify the utility of the platform, fluctuations in pH had minimal impact on growth and productivity. An increase in lactate production was observed which matched observations from similar literature studies.^45^ The impact of DOT fluctuations was more pronounced; a 35% decrease in growth and product titre was observed with the highest growing cell line, as well as significant lactate accumulation. In terms of product quality, two cell lines subjected to DOT fluctuations showed a slight increase in G0F glycans and decrease in G1F + G2F whereas a further cell line showed the opposite effect. This variation in response is consistent with other studies^53^ although most of these were performed under non‐fluctuating conditions.

Overall, this work suggests that the impact of pH and DOT fluctuations are cell line and product specific and are more likely to affect higher growing cell lines. The automated miniature bioreactor therefore provides a platform for rapid investigation of potential large‐scale culture heterogeneities on candidate cell lines and antibody products. Data from fluctuating and non‐fluctuating bioreactor studies is currently being used to try and predict overall culture performance at large scale using population balance approaches.

## AUTHOR CONTRIBUTIONS

**Roman Zakrzewski:** Conceptualization (lead); data curation (lead); formal analysis (lead); investigation (lead); methodology (lead); writing – original draft (lead); writing ‐ review and editing (equal). **Gary J. Lye:** Conceptualization (supporting); supervision (supporting); writing – review and editing (equal). **Kenneth Lee:** Conceptualization (supporting); data curation (supporting); supervision (supporting); writing – review and editing (supporting).

### PEER REVIEW

The peer review history for this article is available at https://publons.com/publon/10.1002/btpr.3264.

## Data Availability

The data that support the findings of this study are available from the corresponding author upon reasonable request.
